# Unmasking Neuroendocrine Prostate Cancer with a Machine Learning-Driven Seven-Gene Stemness Signature That Predicts Progression

**DOI:** 10.3390/ijms252111356

**Published:** 2024-10-22

**Authors:** Agustina Sabater, Pablo Sanchis, Rocio Seniuk, Gaston Pascual, Nicolas Anselmino, Daniel F. Alonso, Federico Cayol, Elba Vazquez, Marcelo Marti, Javier Cotignola, Ayelen Toro, Estefania Labanca, Juan Bizzotto, Geraldine Gueron

**Affiliations:** 1 Departamento de Química Biológica, Facultad de Ciencias Exactas y Naturales, Universidad de Buenos Aires, Buenos Aires C1428EGA, Argentina; asabater@qb.fcen.uba.ar (A.S.);; 2 Instituto de Química Biológica de la Facultad de Ciencias Exactas y Naturales (IQUIBICEN), CONICET-Universidad de Buenos Aires, Buenos Aires C1428EGA, Argentina; 3 Instituto de Tecnología (INTEC), Universidad Argentina de la Empresa (UADE), Buenos Aires C1073AAO, Argentina; 4 Department of Genitourinary Medical Oncology and The David H. Koch Center for Applied Research of Genitourinary Cancers, The University of Texas MD Anderson Cancer Center, Houston, TX 77030, USA; 5 Centro de Oncología Molecular y Traslacional y Plataforma de Servicios Biotecnológicos, Departamento de Ciencia y Tecnología, Universidad Nacional de Quilmes, Bernal B1876BXD, Argentina; 6 Sector de Oncología Clínica, Hospital Italiano de Buenos Aires, Buenos Aires C1199ABB, Argentina

**Keywords:** prostate cancer, stemness, gene signature, prognosis, machine learning, neuroendocrine transdifferentiation, large cell neuroendocrine carcinoma

## Abstract

Prostate cancer (PCa) poses a significant global health challenge, particularly due to its progression into aggressive forms like neuroendocrine prostate cancer (NEPC). This study developed and validated a stemness-associated gene signature using advanced machine learning techniques, including Random Forest and Lasso regression, applied to large-scale transcriptomic datasets. The resulting seven-gene signature (*KMT5C*, *DPP4*, *TYMS*, *CDC25B*, *IRF5*, *MEN1*, and *DNMT3B*) was validated across independent cohorts and patient-derived xenograft (PDX) models. This signature demonstrated strong prognostic value for progression-free, disease-free, relapse-free, metastasis-free, and overall survival. Importantly, the signature not only identified specific NEPC subtypes, such as large-cell neuroendocrine carcinoma, which is associated with very poor outcomes, but also predicted a poor prognosis for PCa cases that exhibit this molecular signature, even when they were not histopathologically classified as NEPC. This dual prognostic and classifier capability makes the seven-gene signature a robust tool for personalized medicine, providing a valuable resource for predicting disease progression and guiding treatment strategies in PCa management.

## 1. Introduction

Prostate cancer (PCa) remains one of the most significant health challenges for men globally, with a high incidence and mortality, particularly in advanced stages of the disease [1]. Despite advancements in early detection and treatment, accurately predicting which patients will experience aggressive disease progression remains a major challenge. A critical gap in the management of PCa is the lack of reliable prognostic biomarkers capable of identifying patients at the highest risk of developing more aggressive forms of PCa, such as neuroendocrine prostate cancer (NEPC), a subtype associated with poor prognosis [2,3]. Addressing this gap is essential for improving patient outcomes and guiding effective therapeutic strategies.

To overcome this challenge, we propose the identification of stem-like characteristics within prostate tumors. Cancer stem cells (CSCs) are a subpopulation of cells within tumors that possess the ability to self-renew, differentiate, and drive tumor growth, metastasis, and resistance to conventional therapies [2,4]. These cells have been implicated in the recurrence and progression of PCa, making them critical targets for both prognostic and therapeutic interventions [5]. Moreover, CSCs are believed to contribute to the heterogeneity of PCa, which complicates treatment and highlights the need for more refined biomarkers [6]. However, despite the recognized importance of CSCs, there is still a need for concise and clinically applicable biomarkers, such as transcriptomic signatures, that can reliably point out the presence of stemness traits in prostate tumors and their associated risk of progression.

In addition to their role in driving tumor growth, CSCs are more abundant after the neuroendocrine differentiation of prostate cancer, which results in NEPC [7]. This PCa subtype can arise either de novo or through the transdifferentiation of adenocarcinoma under selective pressures such as androgen deprivation therapy (ADT) [8,9]. This transdifferentiation process, driven by cellular plasticity and epigenetic changes, results in a highly aggressive cancer subtype that is associated with poor outcomes and limited treatment options [10,11]. Identifying biomarkers that can detect early shifts toward a neuroendocrine phenotype is crucial for managing treatment-resistant cases. However, existing NEPC-related gene signatures are often complex, including a large number of genes, limiting their practical use in clinical settings [12,13].

In this study, we aimed to address these challenges by developing a concise and robust stemness-associated gene signature using machine learning techniques. By analyzing large-scale transcriptomics data from multiple cohorts, we identified a seven-gene signature that predicts multiple PCa disease progression events. This signature was rigorously validated across independent datasets and further substantiated using patient-derived xenograft (PDX) models and a NEPC dataset, where we observed that our signature is able to classify samples as NEPC and, particularly, the large cell neuroendocrine carcinoma subtype. Our comprehensive approach provides a novel and clinically applicable tool for patient stratification and treatment personalization, offering new insights into the role of stem-like traits in PCa and their association with neuroendocrine differentiation.

## 2. Results

### 2.1. Dysregulation of Stemness-Associated Genes Across Multiple PCa Comparisons

**Figure 1:**
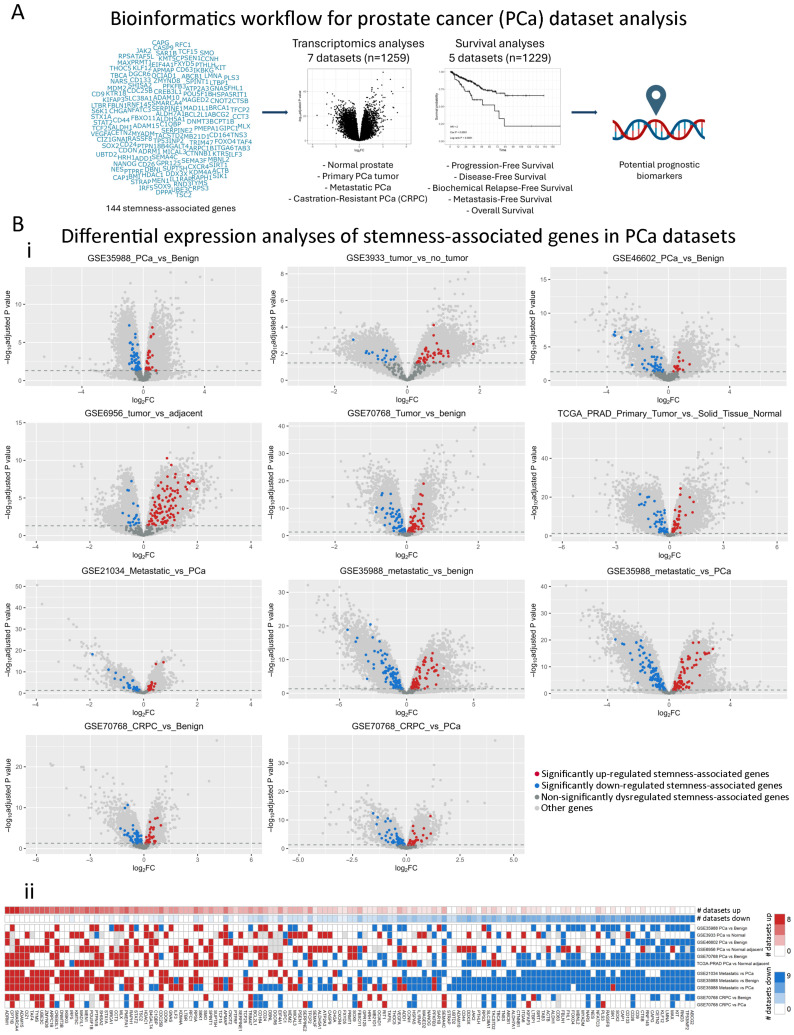
Stemness-associated gene expression changes in PCa patient samples using multiple public datasets. (**A**) Schematic representation of gene selection, transcriptomics, and survival analyses to define potential prognostic biomarkers. (**B**) (**i**) Volcano plots showing the results of the differential expression analyses of all available genes within the included transcriptomics datasets. Red = significantly upregulated stemness-associated gene. Blue = significantly downregulated stemness-associated gene. Dark gray = Non-significantly dysregulated stemness-associated genes. Light gray = other genes available in the dataset. (**ii**) Summary heatmap of the transcriptomics analyses performed in multiple publicly available datasets (n = 1259). Genes of interest and the results of the differential expression analysis for each dataset are displayed. Each row represents the results of a specific comparison. Annotation depicts the absolute number (#) of comparisons in which each gene is upregulated (red) or downregulated (blue). Red = significantly upregulated gene. Blue = significantly downregulated gene. White = not significant changes. Gray = not available. Datasets: GSE35988 (n = 122); GSE3933 (n = 103); GSE46602 (n = 50); GSE6956 (n = 87); GSE70768 (n = 179); TCGA-PRAD (n = 548); GSE21034 (n = 150). Statistical significance was set at adjusted *p*-value < 0.05.

We gathered 144 stemness-associated genes in PCa from the literature [6,14,15,16,17,18] (Supplementary Table S1) and analyzed their expression and association with multiple survival endpoints (Figure 1A). First, we performed pairwise differential gene expression analyses using 7 PCa datasets (n = 1259), which included 11 comparisons between normal prostate, primary tumor, metastatic, and castration-resistant PCa (CRPC) samples (Figure 1A). Volcano plots evidenced the dysregulation of 139 stemness-associated genes, with both upregulation (red, adjusted *p* < 0.05, log2FC > 0) and downregulation (blue, adjusted *p* < 0.05, log2FC < 0) in all comparisons (Primary PCa vs. Benign/Normal/Adjacent, Metastatic PCa vs. Benign, Metastatic PCa vs. Primary PCa, CRPC vs. Benign; CRPC vs. Primary PCa) (Figure 1(Bi)). Figure 1(Bii) summarizes these results across comparisons. We observed that all stemness genes were dysregulated in at least one dataset, with 29 genes consistently upregulated and 26 genes consistently downregulated (Figure 1(Bii), Supplementary Table S2).

### 2.2. Association of Stemness Markers with PCa Patients’ Survival

**Figure 2:**
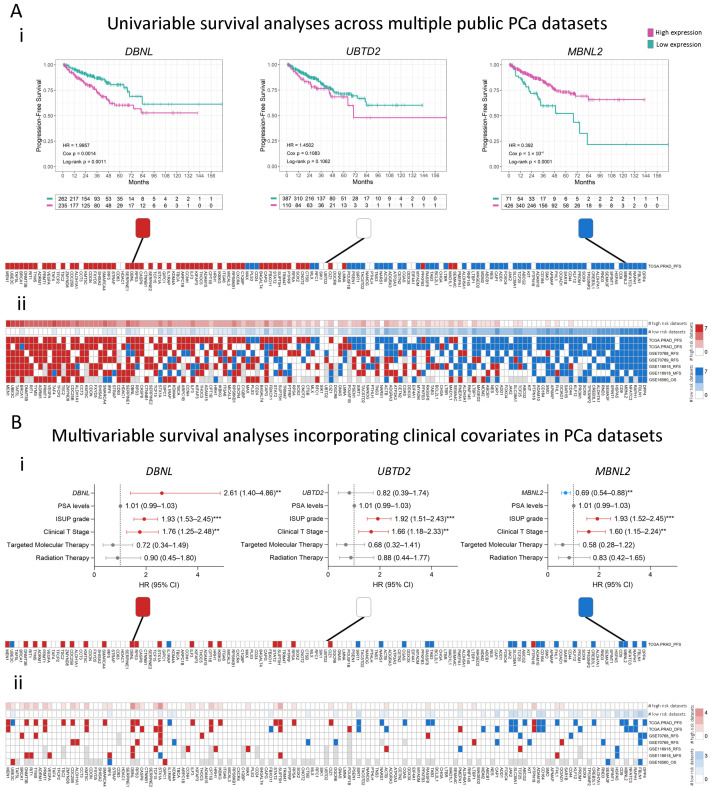
Uni and multivariable survival analysis. (**A**) (**i**) Examples of Kaplan–Meier (KM) curves depicting the association of each gene to the risk of event (purple = high expression of a gene; green: low expression of a gene). HR: Hazard Ratio; Cox *p*: *p*-value from the Cox proportional hazards model. Log-rank *p*: *p*-value of the log-rank test. (**ii**) Summary heatmap of the univariable survival analyses performed on multiple datasets. (**B**) (**i**) Examples of forest plots depicting the association of each gene to the risk of event adjusted for all available covariates using the TCGA-PRAD dataset. (**ii**) Summary heatmap of the multivariable survival analyses performed on multiple datasets. Red boxes indicates that high gene expression is associated with a higher risk of an event (HR > 1 and Cox *p* < 0.05), blue boxes indicate that high gene expression is associated with a lower risk of survival-related events (HR < 1 and Cox *p* < 0.05), and white boxes indicate that there are no significant associations between gene expression and risk of an event. Gray = gene not available. Patients were stratified by the optimal cutoff for each gene, calculated using the Cutoff Finder tool. All comparisons consider low-expression patients as the reference group. Annotation depicts the absolute number (#) of comparisons in which high expression of each gene is associated with high (red) or low (blue) risk. OS: overall survival; DSS: disease-specific survival; PFS: progression-free survival; RFS: relapse-free survival; MFS: metastasis-free survival. Datasets: TCGA-PRAD (n = 497 PFS, n = 337 DFS); GSE70768 (n = 111 RFS); GSE70769 (n = 92 RFS); GSE116918 (n = 248 RFS and MFS); GSE16560 (n = 281 OS). Statistical significance was set at Cox *p* < 0.05. ** Cox *p* < 0.01; *** Cox *p* < 0.001.

We evaluated the association with different survival events in PCa patients, including progression-free survival (PFS), biochemical relapse-free survival (RFS), metastasis-free survival (MFS), overall survival (OS), and disease-specific survival (DSS) for the 139 differentially expressed genes. Figure 2(Ai) shows representative Kaplan–Meier plots for three example genes (*DBNL*, *UBTD2*, and *MBNL2*) in the TCGA-PRAD dataset (n = 497, PFS). The results showed that high expression of *DBNL* and low expression of *MBNL2* were significantly associated with poor PFS (HR = 2, Log-rank *p* = 0.0011 and HR = 0.39, Log-rank *p* < 0.0001, respectively, Figure 2(Ai), left and right panels). No significant associations were observed for *UBTD2* (Log-rank *p* = 0.1062, Figure 2(Ai), middle panel). Figure 2(Aii) shows a heatmap summarizing the results of the univariable survival analysis for each of the 139 candidate genes performed across the 5 training datasets including 5 different types of events (n = 1229; detailed in Supplementary Table S3). The results are color-coded as follows: red squares represent genes with high expression significantly associated with shorter times to the event, white squares indicate genes with no significant associations to the event, and blue squares represent genes with high expression significantly associated with a better outcome (longer times to the event). Of note, there was a group of genes whose high expression was consistently associated with poor prognosis (Figure 2(Aii), left, in red), while others were associated with a better outcome (Figure 2(Aii), right, in blue).

Next, we performed multivariable Cox regression analyses for each of the 139 previously mentioned genes to evaluate their independence from other known risk factors for PCa progression in predicting an event. For the three examples mentioned above, *DBNL* (HR = 2.61, 95% CI 1.40–4.86, Cox *p* = 0.002) and *MBNL2* (HR = 0.69, 95% CI 0.54–0.88, Cox *p* = 0.003) displayed a significant association with high and low risk of PFS, respectively, independently from the other covariates available in the TCGA-PRAD dataset (PSA levels, ISUP grade, Clinical T Stage, and Targeted Molecular/Radiation Therapy; Figure 2(Bi)). No significant associations were observed for *UBTD2* (Figure 2(Bi)). The overall results for the multivariable survival analyses are summarized as a heatmap in Figure 2(Bii) and detailed in Supplementary Table S4. Most associations observed in the univariable survival analysis (Figure 2(Aii)) lost statistical significance after adjusting for clinical covariates (Figure 2(Bii)).

### 2.3. Modeling a Stemness-Associated Signature with Prognostic Value

**Figure 3:**
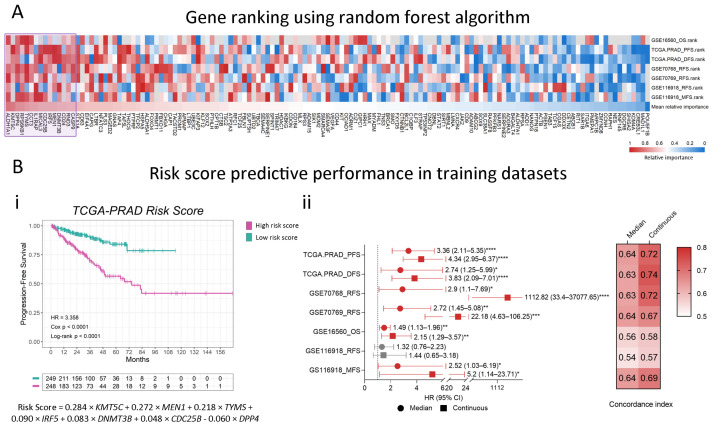
Machine learning Random Forest algorithm for prognostic candidates’ selection. (**A**) Heatmap summarizing the relative importance of the variables (genes) for all training datasets. The relative importance was converted into percentiles, where 1 represents maximum relative importance (red) and 0 indicates minimum relative importance (blue). Gray = gene not available in the dataset. The 15 top-ranked genes (purple box) were selected as candidates for our stemness-associated risk signature. (**B**) (**i**) Example of Kaplan–Meier (KM) curve using the TCGA-PRAD dataset depicting the association of the seven-gene score to the risk of progression (purple = high seven-gene score; green: low seven-gene score). The coefficients for each gene were calculated by Lasso regression using TCGA-PRAD data, and the seven-gene score was constructed as follows: 0.284 × *KMT5C* + 0.272 × *MEN1* + 0.218 × *TYMS* + 0.090 × *IRF5* + 0.083 × *DNMT3B* + 0.048 × *CDC25B* − 0.060 × *DPP4*. Patients were stratified by the median of the score. HR: Hazard Ratio; *p*-value: *p*-value from the Cox proportional hazards model. Log-rank *p*: *p*-value of the Log-rank test. (**ii**) Summary forest plot displaying the survival analysis of the association of the seven-gene signature with the risk of disease-progression events in the training datasets. Patients’ survival was analyzed by either stratification by the median of the seven-gene score (circles) or taking the seven-gene score as a continuous variable (squares). Red corresponds to statistically significant associations (Cox *p* < 0.05) and gray corresponds to not significant associations. On the right, heatmap depicting the concordance index value for each of the analyses. The concordance index is a performance measure of the signature within each dataset. Cox *p*: *p*-value of the Cox regression coefficient. HR = Hazard Ratio. (95% CI) = 95% Confidence Interval. PFS: progression-free survival; DFS: disease-free survival; RFS: relapse-free survival; OS: overall survival; MFS: metastasis-free survival. Statistical significance was set at Cox *p* < 0.05 (red). * Cox *p* < 0.05; ** Cox *p* < 0.01; *** Cox *p* < 0.001; **** Cox *p* < 0.0001.

We used a machine learning algorithm to identify the most relevant prognostic candidate genes to model a gene-expression signature that could stratify patients into risk groups for disease progression and death. We used a Random Forest algorithm to rank genes according to their relevance for event prediction in the training datasets and calculated the mean relative importance score for each gene (Figure 3A). The top 15 genes were *ALDH1A1*, *KMT5C*, *DPP4*, *RPS6KB1*, *TYMS*, *CCT3*, *IL1RAP*, *MICAL3*, *CDC25B*, *IRF5*, *MEN1*, *DNMT3B*, *CD24*, *RND3*, and *CASP9* (Figure 3A, purple square). Next, we used these genes to develop our stemness-associated risk signature. Model coefficients were calculated on the TCGA-PRAD cohort by Lasso regression, a feature selection method that keeps the most important predictors by shrinking the coefficients of less significant genes to zero. This analysis resulted in a signature of seven significant genes, generating the following weighted linear model:

0.284 × *KMT5C* + 0.272 × *MEN1* + 0.218 × *TYMS* + 0.090 × *IRF5* + 0.083 × *DNMT3B* + 0.048 × *CDC25B* − 0.060 × *DPP4*, (1)

where gene expressions are considered a continuous variable (Figure 3(Bi)).

Next, in order to evidence this seven-gene signature prognostic performance, we calculated the risk score for each patient on the TCGA-PRAD dataset and stratified them into high-risk and low-risk groups using the median score as the cut point. As expected, we evidenced a shorter progression time for the high-risk group compared to the low-risk group (HR = 3.36, 95% CI 2.11–5.35, Log-rank *p* < 0.0001, Figure 3(Bi)). When we considered the score as a continuous variable, we observed an HR = 4.34 (95% CI 2.95–6.37, Cox *p* < 0.0001) for each unit increase in the score (Figure 3(Bii), Supplementary Table S5). We then extended the score to the other training datasets and corroborated its prognostic significance within these cohorts. When analyzing the event-free survival in all the other training datasets, we observed significant associations with our model in five out of six analyses, both using the dichotomous and continuous score, suggesting that our seven-gene signature is able to predict the risk of multiple disease-progression events across our training cohorts (Figure 3(Bii), Supplementary Table S5). The identified genes and the developed risk score model effectively stratify patients based on their risk of adverse outcomes, suggesting their potential as prognostic biomarkers.

### 2.4. Consistent Performance Across Validation Datasets

**Figure 4:**
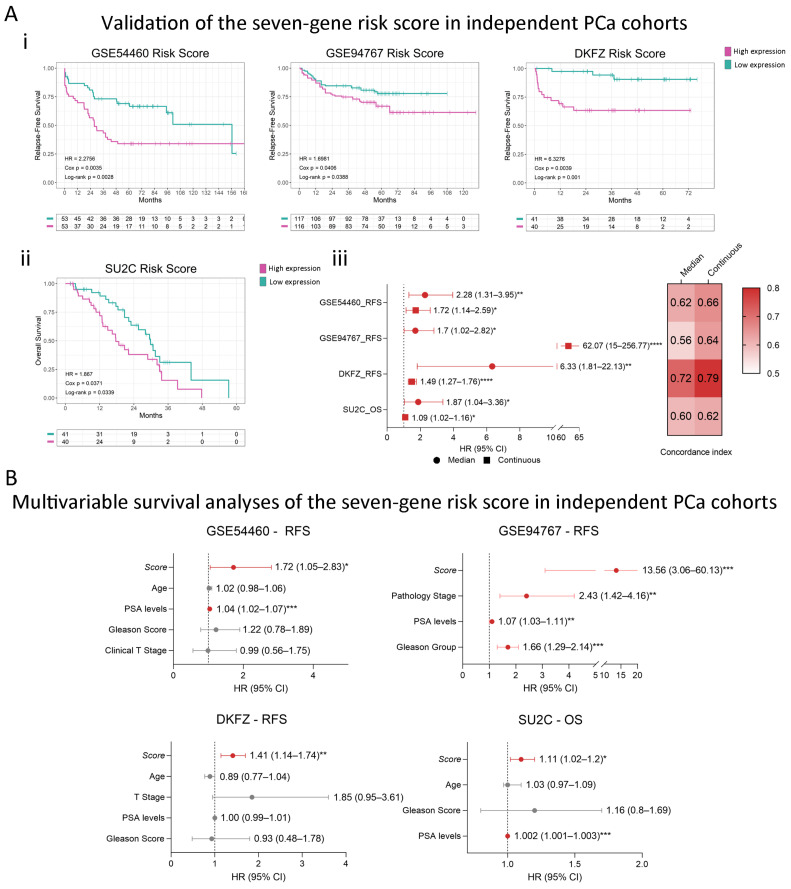
Gene signature’s performance across external validation datasets. (**A**) (**i**) Kaplan–Meier curves depicting the association of the seven-gene score to the risk of disease-progression events included in the validation datasets. (**ii**) Kaplan–Meier curve depicting the association of the seven-gene score to the risk of death of metastatic PCa patients from the SU2C dataset. The coefficients for each gene were calculated by Lasso regression using TCGA-PRAD data, and the seven-gene score was calculated as follows: 0.284 × *KMT5C* + 0.272 × *MEN1* + 0.218 × *TYMS* + 0.090 × *IRF5* + 0.083 × *DNMT3B* + 0.048 × *CDC25B* − 0.060 × *DPP4*. Patients were stratified by the median of the score. HR: Hazard Ratio; Cox *p*: *p*-value from the Cox proportional hazards model. Log-rank *p*: *p*-value of the Log-rank test. (**iii**) Summary forest plot displaying the survival analysis of the association of the seven-gene signature with the risk of disease-progression events in the validation datasets. Patients’ survival was analyzed by either stratification by the median of the seven-gene score (circles) or taking the seven-gene score as a continuous variable (squares). On the right, heatmap depicting the concordance index value for each of the analyses. The concordance index is a performance measure of the signature within each dataset. RFS: relapse-free survival; OS: overall survival. (**B**) Forest plots depicting the association of each gene to the risk of event adjusted for all available covariates within each validation dataset. Red corresponds to statistically significant associations (Cox *p* < 0.05) and gray corresponds to not significant associations. Cox *p*: *p*-value of the Cox regression coefficient. HR = Hazard Ratio [95% CI] = 95% Confidence Interval. Datasets: GSE54460 (n = 106); GSE94767 (n = 233); DKFZ (n = 81); SU2C-PCF (n = 81). Statistical significance was set at Cox *p* < 0.05 (red). * Cox *p* < 0.05; ** Cox *p* < 0.01; *** Cox *p* < 0.001; **** Cox *p* < 0.0001.

Next, we validated our model using datasets from independent cohorts (n = 501). We calculated the seven-gene score for all patients in the different datasets and categorized them into high or low risk using the median value as a cutoff. Interestingly, the risk score was significantly associated with event-free survival in all validation cohorts (Figure 4(Ai,Aii)). Of note, in the SU2C dataset, which comprises metastatic PCa samples, patients with high scores had nearly 2-fold higher risk of death compared to patients with low scores (Figure 4(Aii)). This demonstrates that the seven-gene signature is a robust predictor of the risk of death even in advanced stages. Moreover, when analyzing the seven-gene signature as a continuous variable, all datasets presented significant results, with higher concordance indexes than the dichotomized analysis (Figure 4(Aiii)). Multivariable analyses demonstrated that our score predicts disease-progression events independently of the other clinicopathological variables (Figure 4B), which highlights its potential utility in clinical decision making.

### 2.5. The Stemness-Associated Gene Signature Captures Neuroendocrine Disease Heterogeneity in the MDA PCa PDX Series

**Figure 5:**
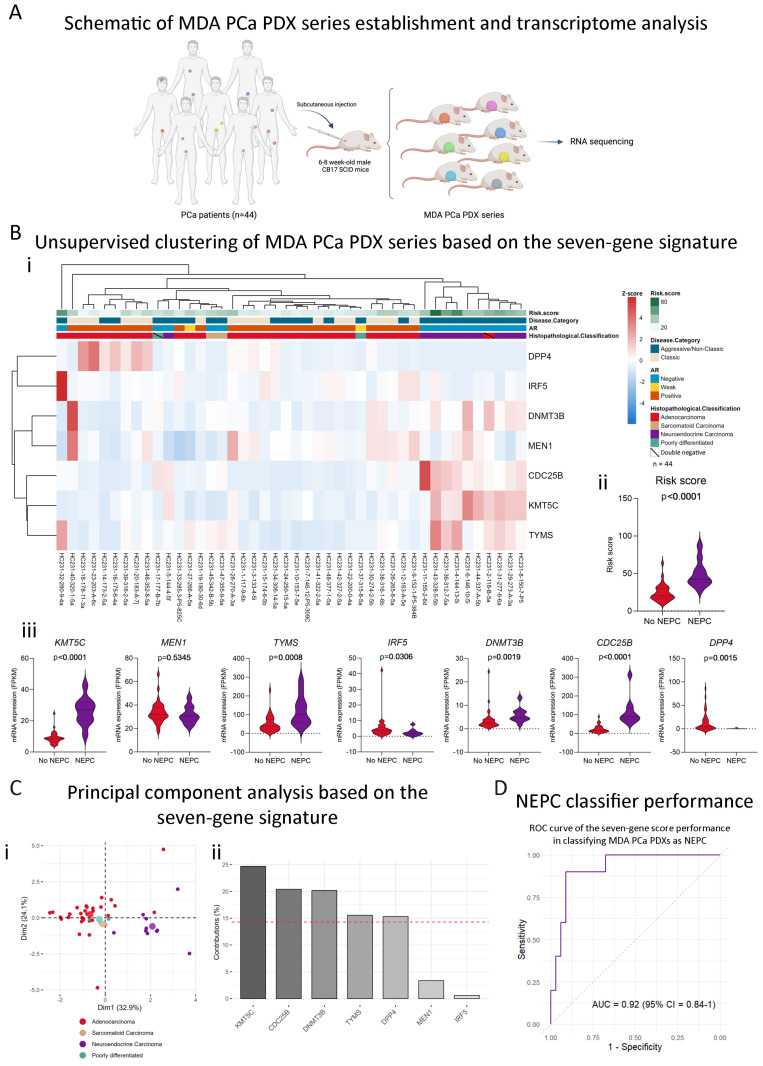
Transcriptomic analysis of the MDA PCa PDX series. (**A**) Schematic representation of the MDA PCa PDX series establishment and transcriptome analysis (n = 44) (created with BioRender.com). (**B**) (**i**) Heatmap depicting unsupervised clustering analysis of RNAseq data from the 44 MDA PCa PDX series considering the expression of the seven-gene signature (*KMT5C*, *MEN1*, *TYMS*, *IRF5*, *DNMT3B*, *CDC25B*, and *DPP4*). Red, white, and blue represent greater, intermediate, and lower gene expression levels. (**ii**) Violin plot showing the seven-gene score levels in no-NEPC and NEPC samples from the MDA PCa PDX series. (**iii**) Violin plots showing the expression levels (FPKM) of the genes included in the seven-gene score in no-NEPC and NEPC samples from the MDA PCa PDX series. (**C**) (**i**) PCA biplot considering the expression of the seven-gene signature using the MDA PCa PDX data assessed by RNA-seq. Each point represents one PDX. Samples are colored according to the histopathological classification: adenocarcinoma (red), sarcomatoid (beige), and neuroendocrine (purple). (**ii**) Bar plot showing the contribution (%) of each gene in the signature to the variance in the PC1 from the PCA. The red dashed line depicts the expected average contribution if all genes weighed the same (value = 14.29%). (**D**) ROC curve showing the performance of the seven-gene score in classifying MDA PCa PDX series as NEPC. 95% CI = 95% Confidence Interval. Statistical significance was calculated using Mann–Whitney test and was set at *p* < 0.05.

Next, we sought to analyze the association between the seven-gene signature and other clinicopathological characteristics available in the MDA PCa PDX series, which was developed in the Laboratory of Dr. Navone within the “Prostate Cancer Patient Derived Xenograft Program” at MD Anderson Cancer Center and the David H. Koch Center for Applied Research of Genitourinary Cancers. PCa tissue samples used for PDX development were derived from therapeutic or diagnostic procedures, namely, radical prostatectomies, orthopedic and neurosurgical procedures to palliate complications, and biopsies of metastatic lesions [19] (Figure 5A). We analyzed the expression of the seven stemness-associated genes selected in the present study using previously generated RNA-Seq data from the 44 MDA PCa PDX series [20]. Surprisingly, the expression of this signature was able to accurately cluster PDXs according to their histopathological classification (adenocarcinoma or sarcomatoid vs. neuroendocrine tumors) in an unsupervised clustering analysis (Figure 5(Bi)). Moreover, NEPC PDXs displayed significantly higher scores (Figure 5(Bii)). Specifically, *CDC25B*, *TYMS*, *KMT5C*, and *DNMT3B* were significantly upregulated in NEPC vs. no-NEPC PDXs, while *IRF5* and *DPP4* were significantly downregulated (Figure 5(Biii)).

These results were also observed in a Principal Component Analysis (PCA) (Figure 5(Ci)), which highlighted *KMT5C* as the main gene in the signature contributing to the variance (PC1) between samples of different histopathological profiles (Figure 5(Cii)), followed by *CDC25B* and *DNMT3B*. Of note, *KMT5C* is also the gene that weighs higher in our score (biggest coefficient, Figure 3(Bi)). To evaluate the power of the signature in predicting whether a tumor is NEPC, we performed receiver operating characteristic (ROC) analysis. The AUC of our seven-gene score was 0.92 (95% CI = 0.84–1) (Figure 5D), highlighting its high performance for classifying NEPC samples.

### 2.6. Our Stemness Score Adds Value to Pre-Existing NEPC Score

**Figure 6:**
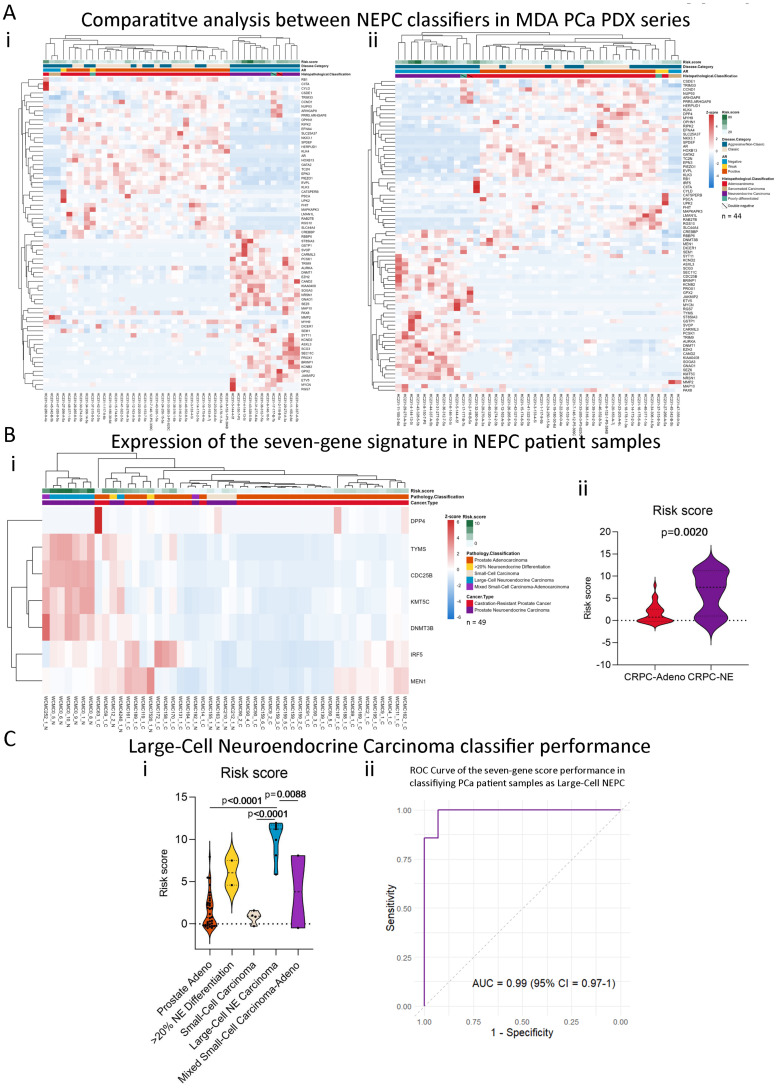
Clinical validation in NEPC samples. (**A**) (**i**) Heatmap depicting an unsupervised clustering analysis of RNAseq data from the MDA PCa PDX series considering the expression of the 70-gene signature proposed by Beltran et al. [12]. (**ii**) Heatmap depicting an unsupervised clustering analysis of RNAseq data from the MDA PCa PDX series considering the expression of the 70-gene signature proposed by Beltran et al. plus the 7 genes (*KMT5C*, *MEN1*, *TYMS*, *IRF5*, *DNMT3B*, *CDC25B*, and *DPP4*) from the risk score model proposed in our work. (**B**) (**i**) Heatmap depicting an unsupervised clustering analysis of RNAseq data from human patients in Beltran et al., dataset (n = 49) [12] considering the expression of the seven-gene signature. Red, white, and blue represent greater, intermediate, and lower gene expression levels. Expression values are presented as z-scores. (**ii**) Violin plot showing seven-gene score levels in Castration-Resistant Prostate Cancer-Adenocarcinoma (CRPC-Adeno) and CRPC-Neuroendocrine (NE) samples from the Beltran et al., dataset. (**C**) (**i**) Violin plot showing risk score levels in samples from the Beltran et al., dataset according to the histological classification: prostate adenocarcinoma without NE differentiation, prostate adenocarcinoma with NE differentiation >20%, small-cell carcinoma, large-cell NE carcinoma, and mixed small-cell carcinoma–adenocarcinoma. (**ii**) ROC curve showing the performance of the seven-gene score in classifying PCa patient samples from Beltran et al.’s dataset as Large-Cell NEPC. 95% CI = 95% Confidence Interval. Statistical significance was calculated using Mann–Whitney test or ANOVA followed by Tukey’s test and was set at *p* < 0.05.

To compare our risk score performance with a pre-established NEPC classification score, we analyzed the expression of the genes from the 70-gene signature by Beltran et al. [12], in the MDA PCa PDX series. We observed a good segregation of the PDXs according to their histopathological classification when using the 70 genes from Beltran et al. NEPC score [12]; however, the 2 double-negative tumors (negative for AR and NE features) were clustered within the NEPC tumors group (Figure 6(Ai)). Nonetheless, when also including the expression of the seven genes identified in this work alongside the genes from the NEPC classification score [12], clustering of the PDX was more accurate, not only grouping adenocarcinomas vs. NEPC tumors but also sarcomatoid samples (Figure 6(Aii)).

### 2.7. The Seven-Gene Signature Effectively Classifies Large-Cell Neuroendocrine Carcinomas

To validate the association of our risk score model with NEPC, we analyzed the transcriptomics dataset from Beltran et al. (n = 49) [12], which includes 15 samples from CRPC-neuroendocrine (NE) and 34 CRPC-adenocarcinomas tumors. Our signature was able to distinguish CRPC-NE tumors to a limited extent (Figure 6(Bi)), while, overall, our risk score was significantly higher in CRPC-NE compared to CRPC-Adeno (*p* < 0.01, Figure 6(Bii)). However, we looked further into the available pathology classification (prostate adenocarcinoma with no neuroendocrine differentiation, n = 34; prostate adenocarcinoma with neuroendocrine differentiation >20%, n = 2; small-cell carcinoma n = 4; large-cell neuroendocrine carcinoma, n = 7; mixed small-cell carcinoma–adenocarcinoma, n = 2) and observed that six out of seven samples of the large-cell NEPC clustered together (Figure 6(Bi)), while the seven-gene signature was particularly higher in that subtype (Figure 6(Ci)). Strikingly, the AUC = 0.99 (95% CI = 0.97–1) suggests that the signature of seven stemness-associated genes proposed in this work is accurate in classifying samples as large-cell NEPC (Figure 6(Cii)). Since large-cell NEPC molecular characterization remains elusive [21], our findings set the grounds for future research on the implications of these genes in this subtype pathogenesis.

## 3. Discussion

In this study, we identified and validated a novel seven-gene signature that represents a significant advancement in the prediction of poor outcomes and molecular detection of NEPC. Our findings demonstrate that this signature not only reliably stratifies PCa patients based on their risk of progression but also reveals a crucial link between stemness-associated pathways and neuroendocrine characteristics. Importantly, this signature is particularly adept at identifying tumors within the Prostate Cancer Foundation (PCF) and World Health Organization (WHO)-defined large-cell neuroendocrine carcinoma [22,23], which is regarded as very rare and associated with very poor outcomes (mean survival of 7 months) [23].

Large-cell NEPCs are high-grade tumors that usually develop from treatment-resistant clones [24]; they are mainly diagnosed histopathologically, thus remaining a challenge and underrecognized [21,22,25]. Hence, there is a need for molecular biomarkers that could subclassify NEPC tumors for better clinical management [21]. The ability of our seven-gene signature to pinpoint this specific aggressive and challenging NEPC subtype underscores the clinical utility of our model in guiding more precise therapeutic interventions.

Our stemness-associated signature addresses a critical need for improving PCa prognosis, while also offering the precise stratification of NEPC, which is often characterized by poor clinical outcomes and high proliferative indices [26]. NEPC is recognized as one of the most aggressive and treatment-resistant forms of PCa, often arising in the context of advanced CRPC after multiple rounds of ADT [27]. While most NEPC cases develop in patients with a history of extensive anti-androgen treatment, the disease can also manifest de novo, albeit rarely, in treatment-naïve patients [9,12]. Further, ADT-induced NE transdifferentiation could be explained by altered mast cell infiltration [28,29]. Maimaitiyiming et al. established a mast cell gene signature with prognostic efficacy in PCa [30], and, interestingly, mast cells have been reported to support the stem phenotype of cancer cells [31]. Altogether, focusing on stemness-associated genes could offer insights into NEPC biology and potential targets.

The molecular landscape of NEPC has been increasingly clarified in recent years, with significant contributions from studies like those of Beltran et al., who delineated the heterogeneity within NEPC and highlighted distinct molecular subtypes [12,22,32]. Their research highlights the genetic, epigenetic, and molecular diversity of NEPC, particularly noting alterations such as RB1 and TP53 loss, MYCN overexpression, and the activation of the PI3K/AKT pathway, which contribute to the aggressive nature of these tumors [12,22,32]. Our study builds on these findings by focusing on a seven-gene stemness signature. Unlike previous signatures that include a broad array of genes, our streamlined seven-gene model achieves comparable or superior predictive accuracy, underscoring its practical utility in diverse clinical contexts.

The biological relevance of the genes in our signature—*KMT5C*, *DPP4* (also known as *CD26*), *TYMS*, *CDC25B*, *IRF5*, *MEN1*, and *DNMT3B*—lies in their involvement in critical processes such as chromatin modification, DNA methylation, DNA repair, cell-cycle regulation, immune escape, and extracellular matrix remodeling [33,34,35,36,37,38,39]. These processes are fundamental to maintaining the plasticity and adaptability of cancer stem cells (CSCs) [5,40], which are enriched after the transdifferentiation of prostate adenocarcinoma into more aggressive neuroendocrine phenotypes [41]. For example, *KMT5C*, *DNMT3B*, and *MEN1* play pivotal roles in chromatin remodeling and methylation [33,34,35], processes that are crucial for the epigenetic reprogramming observed in NEPC [42]. Additionally, *TYMS* has been previously associated with neuroendocrine differentiation in other types of cancer [43,44]. The integration of these stemness-associated genes into our model highlights the potential for characterizing NEPC-like tumors.

One of the key strengths of our study is the extensive validation of our signature across patient datasets and PDX models. The latter, which faithfully replicate the histological and genetic features of human tumors, are widely regarded as the gold standard for preclinical studies [45]. Our findings demonstrate that the seven-gene signature consistently distinguishes NEPC from other PCa subtypes in these models, underscoring its clinical utility and potential for identifying NEPC-like tumors. This aspect of our research not only validates the predictive power of the signature but also highlights its potential utility in translational research, particularly in the development of novel therapeutic strategies aimed at targeting the molecular underpinnings of NEPC.

## 4. Materials and Methods

### 4.1. Stemness-Associated Genes

We gathered 144 stemness-associated genes from the PCa literature [6,14,15,16,17,18]. We conducted transcriptomics analyses using publicly available PCa datasets (see below) to study differential gene expression across multiple comparisons, including normal/benign tissues, primary PCa tumors, CRPC tumors, and metastatic samples. We performed univariable survival analysis to study the association between gene expression and different endpoints (progression, disease-free time biochemical-relapse, metastasis, and death). We also performed multivariable survival analyses that included clinicopathological features as covariables.

### 4.2. Gene Expression Analyses in Human Patients

#### 4.2.1. Dataset Selection Criteria

To study differential gene expression across different PCa datasets, we searched the Gene Expression Omnibus (GEO, accessed date 1 October 2021) and the Genomic Data Commons Data Portal (accessed date 1 October 2021) to identify eligible datasets that met the following criteria: (1) PCa tissue samples with available transcriptomic and clinicopathological data; (2) datasets with ≥2 different tissue sample types (Table 1).

#### 4.2.2. Differential Gene Expression Analyses

We used the limma package (Linear Models for Microarray Analysis, version 3.58.1) [54] to study differential gene expression from both microarrays and RNA sequencing (RNA-seq). In the case of non-normalized data, quantile normalization was applied [54]. For RNA-seq data, the voom function in the limma package was used for processing [55]. We conducted pair-wise differential expression analyses within each dataset. For each available probe or gene, the fold changes (FCs) between conditions were calculated and expressed as log2FC. To correct for multiple testing, we used the Benjamini-Hochberg method to control the type I error and reported adjusted *p*-values.

### 4.3. Association Between Gene Expression and Patients’ Outcomes

#### 4.3.1. Dataset Selection Criteria

To perform survival analysis, we searched the Gene Expression Omnibus (GEO) (accessed date 1 October 2021), cBioPortal (accessed date 1 October 2021), and the Genomic Data Commons Data Portal (accessed date 1 October 2021) to identify eligible datasets that met the following criteria: (1) PCa cases with available gene expression data and (2) available clinicopathological features with ≥5 years of follow-up. Gene expression and clinical data were downloaded and analyzed for the resulting selected datasets. Samples with incomplete gene expression data or missing essential clinicopathological metadata were not included. Datasets were randomly distributed in training (5 datasets, 7 survival analyses) and validation cohorts (4 datasets) (Table 2).

#### 4.3.2. Survival Analyses

We used the Log-rank test to analyze differences in the risk of disease-progression events between different groups of patients [62]. To stratify patients according to high or low expression, we used the Cutoff Finder tool to find the optimal cutoff point for each gene [63]. The Cox proportional hazards model was used to estimate the risk of the disease-progression event for the different groups [64]. Multivariable analyses included clinicopathological features as covariables. All modeling, calculations, and graphs were performed with the R packages survival (version 3.7-0) [65] and survminer (version 0.4.9) [66].

### 4.4. Selection of Candidate Genes for Modeling a Risk Score

To identify the 15 most important genes for predicting events, we used a machine learning ensemble-based approach (i.e., the Random Forest Classifier) as implemented in the randomForestSRC R package (version 3.3.1) [67]. The mtry and nodesize parameters were optimized through a grid search approach to minimize the out-of-bag error using the tune function (set.seed(1), ntree = 500), and are included in Supplementary Table S6. We used the Breiman–Cutler variable importance (VIMP) measure to estimate the relative importance of each variable in predicting event-free survival within the training datasets. We applied the subsampling method [68] to estimate the standard error of the VIMP and to calculate the confidence intervals. Genes were ranked according to their variable importance. To facilitate the comparison across datasets, VIMP values were converted into fractions, with 1 representing the most important variable and 0 representing the least important variable within a given dataset.

### 4.5. Gene Signature and Risk Score Calculation

We modeled a risk score based on the gene expression of the 15 most important genes identified across training datasets using Random Forest. To develop this risk score, we calculated model coefficients through Lasso regression using TCGA-PRAD data. Patient scores were then calculated based on the expression of the selected genes following Lasso regression. The performance of this risk score was evaluated within each training dataset. Univariable Cox regression was used to estimate the risk of poor survival in patients with high-risk scores. Patients were stratified either by a dichotomized risk score (with the median as the cut point) or by a continuous risk score. The concordance index (CI) was used to measure the performance of the signature within each dataset.

In the validation stage, those same coefficients were used in all additional datasets. For each patient, the score was calculated, and its association with event-free survival was studied using univariable and multivariable Cox regressions.

### 4.6. Transcriptome Analysis of MDA PCa PDX Series

To assess the association of the stemness signature and other clinicopathological characteristics in an extensively annotated cohort, we used the MDA PCa PDX series, which was previously developed in the “Prostate Cancer Patient Derived Xenograft Program” at MD Anderson Cancer Center and the David H. Koch Center for Applied Research of Genitourinary Cancers [19]. Briefly, PCa tissue samples used were derived from various procedures, and small pieces were then implanted into subcutaneous pockets of 6- to 8-week-old male CB17 SCID mice (Charles River Laboratories) [19]. RNA-Seq and transcriptome analysis on these samples was performed as previously described [20].

### 4.7. Unsupervised Clustering and Principal Component Analysis (PCA)

Unsupervised clustering analysis including the expression data of the stemness-associated genes included in the signature was performed using the pheatmap (version 1.0.12) [69] package, and Principal Component Analysis was performed using the factoextra package (version 1.0.7) in R (version 4.3.0) [70].

### 4.8. Receiver Operating Characteristic (ROC) Curve for NEPC Classification

The pRoc package (version 1.18.5) [71] was used for the estimation of the receiver operating characteristic (ROC) curve and the area under the ROC curve (AUC). Confidence intervals were calculated by the DeLong method [71].

### 4.9. NEPC Patients’ Samples Dataset

To assess gene expression in NEPC samples, we downloaded the data from the Neuroendocrine Prostate Cancer (Multi-Institute, Nat Med 2016) dataset published by Beltran et al. [12] from cBioPortal (accessed date 30 July 2024) [72,73,74]. Briefly, this dataset contains transcriptomics and histopathological data from 49 PCa samples (34 CRPC-Adeno and 15 CRPC-NE) obtained by RNA-Seq.

### 4.10. Statistical Analyses

All bioinformatics analyses were performed using the R programming language (version 4.3.0) [75] through the RStudio platform (RStudio, PBC, Boston, MA, USA, version 2024.04.1) [76]. The tidyverse package (version 2.0.0) was used for general data analysis and manipulation [77]. For graphics, the ggplot2 (version 3.5.1) [78], ggpubr (version 0.6.0) [79], and RColorBrewer (version 1.1-13) [80] packages were used. Datasets available in GEO were downloaded with GEOquery (version 2.70.0) [81]. All heatmaps were created with the pheatmap package (version 1.0.12) [69]. Forest plots were created using GraphPad Prism software (version 8.4.2, La Jolla, CA, USA). The Mann–Whitney test and ANOVA, followed by Tukey’s test, were used to assess differences in risk score and gene expression values across groups. We used the Log-rank test and Cox proportional hazard model regression to study the association between gene expression and patients’ survival. Multivariable analyses were performed in R and plotted in GraphPad Prism software (version 8.4.2, La Jolla, CA, USA). Statistical significance was set at *p* < 0.05.

## 5. Conclusions

This study presents a significant advancement in PCa prognosis and the classification of NEPC, particularly for the challenging large-cell subtype. Importantly, PCa cases presenting this molecular signature, even when not histopathologically identified as NEPC, also exhibit a poor prognosis. This reinforces the clinical relevance of our model, which is capable of identifying aggressive tumor subtypes that may not yet display overt NE differentiation but still represent a high risk for adverse outcomes. Through the development of this novel stemness-associated seven-gene signature, our model offers a robust and practical tool with potential clinical application, paving the way for more personalized and effective therapeutic strategies in PCa.

### Limitations

Despite the robustness of our findings, there are several limitations that must be acknowledged. Our study primarily relies on transcriptomic data from publicly available repositories, which, while comprehensive, may not fully represent the genetic diversity of PCa patients globally. Future research should focus on further validating our signature in ethnically and genetically diverse cohorts to ensure its broad applicability. Further, the analysis is based on retrospective data, and further prospective validation in clinical settings is necessary to confirm its full applicability. Additionally, while our focus on transcriptomic data provided valuable insights into NEPC biology, integrating multi-omics data, including proteomics and metabolomics, could enhance the predictive power of our model. Moreover, the scarce number of NEPC samples with transcriptomics data and, particularly, of large-cell NEPC (likely due to under-recognition and underreporting [21]) requires further validation in larger cohorts. Functional validation of the identified genes through in vivo studies will also be critical for determining their role in disease and translating findings into clinical practice.

## Figures and Tables

**Table 1:** PCa transcriptomics datasets for differential expression analysis.

Dataset	Samples
GSE35988 [46]	Localized PCa (n = 59), matched benign prostate tissues (n = 28), and metastatic CRPC (n = 35).
GSE3933 [47,48]	Localized PCa (n = 62) and normal prostate (n = 41).
GSE46602 [49]	PCa (n = 36) and benign tissue (n = 14).
GSE6956 [50]	Primary PCa (n = 69) and normal adjacent prostate (n = 18).
GSE70768 [51]	Primary PCa (n = 112), benign tissue (n = 74) and CRPC (n = 13).
TCGA-PRAD [52]	Primary PCa (n = 497) and normal adjacent tissue samples (n = 51).
GSE21034 [53]	Primary PCa (n = 131) and metastatic tissue samples (n = 19).

**Table 2:** PCa transcriptomics datasets for survival analyses.

Dataset	Samples	Survival Endpoint	Covariates	Cohort
TCGA-PRAD [52]	497 PCa (RNAseq)	Disease Progression Disease-Free Time (n = 337)	Gleason Group, PSA levels, Clinical T Stage, Targeted Molecular/Radiation Therapy	Training
GSE70768 [51]	111 PCa (Microarray)	Biochemical Relapse	Age, Gleason Group, PSA levels, T Stage	Training
GSE70769 [51]	92 PCa (Microarray)	Biochemical Relapse	Gleason Group, PSA levels, T Stage	Training
GSE116918 [56]	248 PCa (Microarray)	Metastasis Development Relapse	Age, Gleason Score, PSA levels, T Stage	Training
GSE16560 [57]	281 PCa (Microarray)	Death	Age, Gleason Group	Training
GSE54460 [58]	106 PCa (RNA-seq)	Biochemical Relapse	Age, Gleason Score, PSA levels, T Stage	Validation
GSE94767 [59]	233 PCa (Microarray)	Biochemical Relapse	Gleason Group, PSA levels, T Stage	Validation
DKFZ [60]	81 PCa (RNA-seq)	Biochemical Relapse	Age, Gleason Score, PSA levels, T Stage	Validation
SU2C-PCF [61]	81 metastatic CRPC (RNA-seq)	Death	Age, Gleason Score, PSA levels	Validation

## Data Availability

The original contributions presented in the study are included in the article/Supplementary Materials, and further inquiries can be directed to the corresponding author/s. The code used to generate the herein presented results is available at https://github.com/asabater00/stemness_signature_PCa (uploaded on 17 October 2024).

## Supplementary Materials

The supporting information can be downloaded at: https://www.mdpi.com/article/10.3390/ijms252111356/s1.
